# Prediction of early race starts in Norwegian-Swedish Coldblooded Trotters

**DOI:** 10.1186/1751-0147-52-53

**Published:** 2010-09-22

**Authors:** Tobias Revold, Stig Larsen, Carl F Ihler

**Affiliations:** 1 Department of Companion Animal Clinical Sciences, Section for Equine Medicine, Norwegian School of Veterinary Science, Post box 8146 Dep., N-0033 Oslo, Norway; 2 Department of Production Animal Clinical Sciences, Centre for Epidemiology and Biostatistics, Norwegian School of Veterinary Science, Post box 8146 Dep., N-0033 Oslo, Norway

## Abstract

**Background:**

Less than a third of Norwegian-Swedish Coldblooded Trotters (NSCTs) have started racing as three year olds since the year 2000 despite the fact that large sums are paid out as price-money in the three year season. Recruitment races are arranged by the Norwegian Trotting Association (NTA) to stimulate early training. The management of young horses varies considerably and a large majority is reared by amateurs. The aim of the present study was to identify predictors of early race starts in young NSCT horses under field conditions.

**Methods:**

Of the 801 registered NSCT horses born in 2005, 144 were randomly selected by stratified sampling with gender and paternal progeny as stratification factors. All horses were examined clinically. Further data were collected from NTA and by interviews of breeders, owners and trainers. The set of dependent variables consisted of "passed recruitment race", "start in regular race by the end of the three year season" and "start in regular race by the end of October in the four year season". Univariate and logistic regression analyses were performed.

**Results:**

Genetic performance potential, as indicated by best linear unbiased prediction (BLUP) indices, was the major predictor of the three dependent variables despite large variation in management. Dam's index was a better predictor than sire's index. However, the probability of early race starts in a horse with a low genetic performance potential can be increased by a favourable management. Examples of advantageous management factors in the present study were a flat pasture the first summer and early training. Nearly all horses racing in the three or four year seasons had passed a recruitment race in the two year season.

**Conclusions:**

The results confirm the value of the published BLUP index as an important tool for the NSCT breeding program. Recruitment races stimulate early training.

## Background

In Norway and Sweden, Standardbreds and Norwegian-Swedish Coldblooded Trotters (NSCTs) are used for harness-racing. The two breeds compete in separate races, but under similar conditions. Standardbreds have been bred solely for their trotting abilities, whereas Coldblooded Trotters have evolved from a rural carriage and riding horse. Today, both breeds are pure trotting racehorses. This is reflected in the breeding programme for NSCT horses [1].

A typical NSCT racing career has a late onset and a long duration compared to a Standardbred career [The Norwegian Trotting Association (NTA), personal communication]. In a study from Finland, it was found that Finnhorses, which are also Coldblooded Trotters, achieved the highest speeds at high ages, while Standardbreds were faster as four year olds [2]. Large parts of the price money are paid out in races for young horses as early evaluation of racehorses contributes to a shortening of generation intervals, which is desirable for the breeding progress.

Despite substantial financial and physical efforts from trainers, owners and the NTA, only 26% to 31% of NSCTs in Norway have raced one or more times as three year olds since year 2000, compared to 38% to 44% for Norwegian Standardbreds [NTA, personal communication]. A study conducted in 1980-1981 showed that approximately 30% of NSCT horses started in regular trotting races as three year olds [3]. Hence, the fraction of NSCT horses racing in the three year season appears fairly constant. On the other hand, the racing speeds in NSCT races have increased considerably over the last decades [4]. Early start in regular races can be seen as a first barrier and an important step on the way to a successful racehorse.

Less than 35% of harness racing horses in Norway are trained by professional trainers [5]. A far lower number of horses are reared by professionals. Therefore, the management of individual young NSCT horses varies considerably.

Various studies have confirmed a moderate to high heritability of performance traits in racehorses [6]. However, to our knowledge, the impact of various management factors on early race starts in NSCT horses has never been studied.

The aim of the present study was to identify predictors of early race starts in young NSCT horses under field conditions.

## Methods

### Horses

All 801 NSCT foals born in 2005 and registered by the NTA were grouped by sire in connection with a study of muscular development and performance. Groups of less than five horses, representing a total of 82 foals, were excluded from the study. From the remaining sires, 20% of the offspring of each gender were randomly sampled for participation in order to give a representative study sample with regards to the stratification factors. Of the selected horses, 30 owners were unwilling to participate, and 11 horses died before start of the study. Additional horses were sampled according to the same criteria for replacement of the non-responders and drop outs. Seven sampled horses from the three northernmost counties of Norway were for logistic reasons treated as non-responders. The stratified sampling procedure with paternal progeny group and gender as stratification factors resulted in a study sample of 68 colts and 77 fillies. One of the fillies was excluded for logistic reasons at a later time and not replaced. The remaining 144 horses were examined between December 13^th^, 2006 and March 15^th^, 2007. Identification was verified by a microchip-reader as all NSCT foals have a microchip implanted.

Of the original 144 horses, seven were reported dead during the study period. One horse was euthanized before start of any races and was therefore excluded from all statistical analyses. Another horse became diseased and was euthanized at the beginning of the three year season and therefore excluded from statistical analyses regarding the three and four year season. Five horses died later and could therefore have started races. These horses were included in all analyses.

### Data collection

The examination included size measurements as well as evaluation of body condition and conformation of the extremities. Further, a questionnaire-based interview of breeder and owner/trainer was performed and muscle biopsies were sampled. Interviews were carried out on the day of examination or later by a telephone interview and consisted of questions related to the early life and management of the horse, such as diseases, feeding, pasture data and early training. The recorded variables were obtained by the same observer for all horses (Table 1). Muscle characteristics of the study objects will be published separately.

**Table 1:** Variables recorded by examination, interview and records from the Norwegian Trotting Association (NTA) with units, range (continuously distributed variables) and number of observations in each category (categorized variables).

Variables recorded by clinical examination and interview:	Unit/categories:	Range of continuously distributed variables [] or numbers in each category ():
Height (tape measure) corrected to 24 months age	Centimetres	[145 - 170]
Chest circumference	Centimetres	[157 - 200]
Body condition	Normal/Abnormal	(137/7)
Conformational abnormalities	Normal/Abnormal	(129/15)
Age at examination	Months	[17 - 24]
Age at weaning	Months	[4 - 21]
Age at introduction to concentrated feeds	Months	[3 - 22]
Amount of concentrated feeds/day at time of examination	Litres	[0 - 9]
Time spent at pasture 1^st ^summer	Weeks	[8 - 20]
Topography of pasture 1^st ^summer	Flat/Intermediate/Hilly	(28/28/69)
Group size at pasture 1^st ^summer	Horses	[2 - 25]
Number of foals in pasture group 1^st ^summer	Foals	[1 - 10]
Daily time spent in paddocks 1^st ^winter	Hours	[1 - 24]
Time spent at pasture 2^nd ^summer	Weeks	[4 - 20]
Topography of pasture 2^nd ^summer	Flat/Intermediate/Hilly	(34/24/72)
Group size at pasture 2^nd ^summer	Horses	[1 - 25]
Number of yearlings in pasture group 2^nd ^summer	Yearlings	[1 - 17]
History of diseases	Yes/No	(11/127)
Accustomed to sulky before pasture 2^nd ^summer	Yes/No	(60/81)
Horse sold prior to examination	Yes/No	(50/94)
Started training before age 18 months	Yes/No	(48/95)
Trained by professional trainer at time of examination	Yes/No	(37/107)

**Variables obtained from the NTA:**		

Gender	Colt/Filly	(68/76)
Sires BLUP-index	-	[109 - 139]
Dams BLUP-index	-	[92 - 125]
BLUP estimate*	*-*	[105 - 132]
Passed Recruitment race	Yes/No	(92/51)
Raced before end of three year season (2008)	Yes/No	(51/91)
Raced until the 28^th ^of October 2009	Yes/No	(70/72)

*mean of indices of sire and dam

The height was measured with a tape measure by recording the distance from the highest point of the withers to the ground following the chest contour. The recorded height was significantly correlated to age at examination. As the height increased by 0.79 centimetres per month as determined by linear regression, recorded height measurements were corrected up to expected height at 24 months age. As chest circumference and age at examination were not significantly correlated, chest circumference was not corrected.

Body condition was subjectively evaluated and classified as normal or abnormal. The abnormality consisted of both under- and overweight. Conformation of the extremities was recorded as abnormal in cases of obvious angular or flexural limb deformities or small or badly shaped hooves not correctable with a single trimming.

From the interview, history of previous diseases with potential impact on the development of the horse was obtained. Daily amount of concentrated feeds was recorded in litres as this was easy for owners to estimate. The summer pastures were categorised by the breeders/owners/trainers as flat (without hilly areas), intermediate (containing flat and hilly areas) or hilly (without flat areas). In 11 cases, the interview data were incomplete, as contact with the breeder or owner/trainer for the period in question was not achieved. In one horse, height and chest circumference were missing for technical reasons. Ten of the included horses were not kept on pasture the first summer and five horses were not kept on pasture the second summer. Pasture related data are therefore missing for these horses.

In the breeding program for NSCTs, each horse is given an individual best linear unbiased prediction (BLUP) index based on the following traits: 1) The genetic potential for race attendance at the age of three to six years (40% of the total index), and 2) the genetic potential for race performances measured by the traits best recorded racing speed, total earnings and percentage of races placed first or second (60% of the total index) [7]. Since the index contains performance records of the relatives it is not a simple index of individual performance, but rather an expression for the expected genetic potential for performance. BLUP indices are updated yearly. BLUP indices of sires and dams were obtained from NTA. As the accuracy of the index increases with every update, the latest available update, performed in 2008, was used. For the included horses themselves, a BLUP value was estimated by calculating the mean of dam's and sire's index. This value was termed BLUP estimate.

The set of dependent variables in the present study was: "recruitment race passed" ("recruitment race"), "start in regular race by the end of the three year season" ("3-year start") and "start in regular race by the end of October in the four year season" ("total start"). The date was chosen to include the major races in the four year season.

The owner receives token price money if the horse passes a recruitment race, which is arranged by NTA to stimulate early training. Recruitment races are not compulsory, but to start in a regular trotting race on a Norwegian racetrack, the horse must pass a qualification race within an age specific speed limit. Racing records describing the three dependent variables for included horses were obtained from the NTA database [5] on October 28^th ^2009.

### Statistics

Assumed continuously distributed variables are expressed by mean values and 95% confidence intervals (CI) constructed using the Student's procedure [8]. Contingency tables are used for expressing categorized factors and variables [9].

For comparison of groups with regard to assumed continuously distributed variables, analysis of variance (ANOVA) was used [8]. Contingency table analysis was used for comparison of groups regarding categorized variables [9].

Comparison of groups was performed two-tailed and differences considered significant at a level of 5%. In order to obtain an optimal set of independent variables to predict each of the three dependent variables, logistic regression analysis was performed [10]. Both forward and backward procedures for inclusion of variables in the models were used sequentially. Variables with *P*-values larger than 0.10 were excluded. Log odds ratio estimates from logistic regressions are presented in tables. The corresponding receiver operating characteristic (ROC) curves are also presented. Areas under the ROC curves are given with CI.

All analyses were performed using JMP - SAS version 8.1. The expected contribution of an independent variable to the outcome of an average horse in the population is illustrated by the product of the log odds ratio estimate (β_i_) and the mean observation for that variable (${\bar{x}}_{\text{i}}$). This value only makes sense when compared to the other variables in the same model.

## Results

Eleven horses were recorded with previous diseases of potential impact on development of the horse.

BLUP estimate and dam's BLUP index, age-corrected height and amount of concentrated feeds per day at the moment of examination were significantly different for all three dependent variables. Also, the BLUP estimate and the dam's BLUP index were significantly higher in horses trained before the age of 18 months than for the others.

Body condition, history of disease, early training and professional training were significantly different for the dependent variable "recruitment race". Recruitment race also acted as an independent variable, which was significantly different for "3-year start" as well as for "total start". A complete list of independent variables significantly different for at least one of the dependent variables in univariable analyses as well as independent variables included in the logistic regression models is given in Tables 2 and 3.

**Table 2:** Mean with 95% confidence interval of continuously distributed variables that were significantly different (*P *≤ 0,05) for at least one of the three dependent variables "recruitment race", "3-year start" or "total start"

Independent variable	Recruitment race		3-year start		Total start	
	Passed (n = 92)	Not passed (n = 51)	Yes (n = 51)	No (n = 91)	Yes (n = 70)	No (n = 72)
BLUP-estimate	118.72* [117.67 - 119.77]	115.45* [114.00 - 116.90]	118.82* [117.34 - 120.31]	116.82* [115.72 - 117.91]	119.46* [118.27 - 120.66]	115.67* [114.50 - 116.84]

Sire's BLUP-index	126.01* [124.41 - 127.61]	122.53* [120.49 - 124.57]	125.33 [123.15 - 127.52]	124.41 [122.80 - 126.02]	126.24* [124.38 - 128.10]	123.28* [121.53 - 125.01]

Dam's BLUP-index	111.44* [110.22 - 112.65]	108.37* [106.54 - 110.21]	112.31* [110.62 - 114.01]	109.23* [107.94 - 110.52]	112.69* [111.28 - 114.09]	108.06* [106.69 - 109.43]

Age-corrected height (cm)	157.51* [156.73 - 158.28]	155.10* [153.89 - 156.32]	157.87* [156.71 - 159.02]	155.95* [155.11 - 156.78]	157.52* [156.56 - 158.47]	155.80* [154.83 - 156.77]

Age at weaning (months)	6.75* [6.21 - 7.28]	8.02* [6.94 - 9.10]	7.08 [6.21 - 7.95]	7.29 [6.63 - 7.96]	6.93 [6.26 - 7.60]	7.52 [6.70 - 8.33]

Age start conc. feeds (months)	6.13 [5.50 - 6.75]	7.38 [6.08 - 8.67]	5.90* [5.30 - 6.49]	7.00* [6.10 - 7.90]	5.99* [5.35 - 6.62]	7.23* [6.17 - 8.28]

Conc. feeds per day (Litres)	4.24* [3.86 - 4.62]	3.26* [2.73 - 3.79]	4.42* [3.93 - 4.91]	3.58* [3.18 - 3.99]	4.37* [3.94 - 4.80]	3.41* [2.96 - 3.86]

* = (*P *≤ 0.05)

Variables included in logistic regression models are underlined.

**Table 3:** Univariate analysis of categorized variables that were significantly different (*P *≤ 0.05) for at least one of the three dependent variables "recruitment race", "3-year start" or "total start", and/or were included in the logistic regression model for at least one of the dependent variables

Independent variable		Recruitment race		3-year start		**Total start**	
	
	Category	Passed (n = 92)	Not passed (n = 51)	Started (n = 51)	Not started (n = 91)	Started (n = 70)	Not started (n = 72)
Gender	Male	49*	18*	29	37	40*	26*
	Female	43*	33*	22	54	30*	46*

Body condition	Normal	91*	45*	50	85	69	66
	Abnormal	1*	6*	1	6	1	6

Pasture topography 1^st ^summer	Flat	19	9	12	16	13	15
	Intermediate	21	7	8	20	16	12
	Hilly	42	26	25	42	34	33

History of disease	Yes	3*	8*	1	10	3	8
	No	85*	41*	48	77	65	60

Accustomed to sulky before pasture 2^nd ^summer	Yes	43	17	20	40	32	28
	No	47	33	30	49	37	42

Horse sold	Yes	38*	12*	21	29	30	20
	No	54*	39*	30	62	40	52

Started training before age 18 months	Yes	42*	6*	22	26	30*	18*
	No	49*	45*	28	65	39*	54*

Trained by professional trainer	Yes	31*	6*	15	21	21	15
	No	61*	45*	36	70	49	57

Recruitment race passed	Yes	-	-	50*	41*	65*	26*
	No	-	-	1*	50*	5*	46*

* = (*P *≤ 0.05)

Variables included in logistic regression models are underlined.

**Figure 1:**
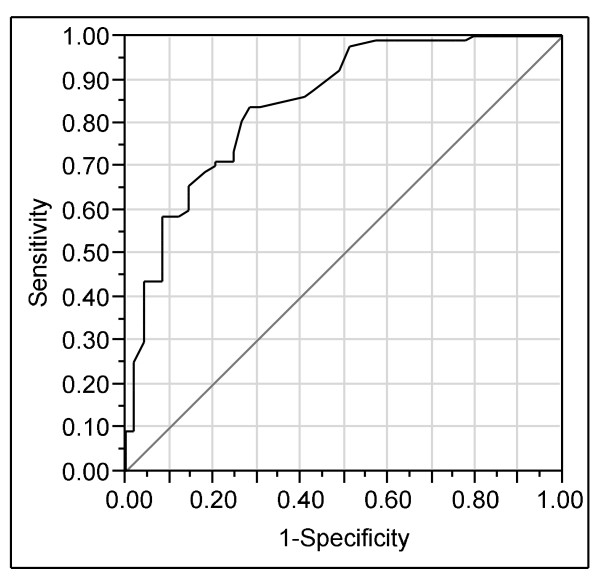
**Receiver operating characteristics (ROC) curve for the logistic regression model predicting not passed "recruitment race"**.

A logistic regression model for prediction of not passed "recruitment race" is given in Table 4 . Negative estimates indicate an increased likelihood of passing. The BLUP index of the dam was the independent variable with the highest contribution to the model. Thus, a horse with a high dam's index was more likely to pass a recruitment race than a horse with a low dam's index. The probability of passing a recruitment race was reduced with a history of previous disease and increased with training by a professional trainer and start of training before the age of 18 months. However, as indicated by the interactions, an age-corrected height above the mean increased the beneficial effect of a professional trainer. Further, the beneficial effect of a professional trainer was reduced if the horse started training early. The ROC-curve for this model is presented in Figure 1 with an area under the ROC curve of 84.7% (CI 84.2 - 85.2%). The area under the ROC curve illustrates the probability that a random horse that had not passed a recruitment race would have a higher test value than a randomly selected horse from the group that passed. Random guessing would give an area under the ROC curve of 50%.

**Table 4:** Log odds ratio estimates ± standard error, mean values and contributions in the logistic regression model for not passed "recruitment race" and interactions accounted for

	Estimate ± SE(β_i_)	Mean value(${\bar{x}}_{\text{i}}$)	Contribution (β_i _* ${\bar{x}}_{\text{i}}$**)**
**Linear contribution**			
Intercept 0	12.77 ± 4.75		
Dam's BLUP-Index	-0.11 ± 0.04	110.34	-12.68
Previous disease (1 = yes)	1.63 ± 0.90	0.08	0.13
Professional trainer (1 = yes)	-3.14 ± 1.03	0.26	-0.81
Trained before 18 months (1 = yes)	-2.93 ± 0.81	0.34	-0.99
			
**Interactions**			
(Corrected height -156.64) * (Professional trainer)	-0.62 ± 0.26		
(Trained before 18 month) * (Professional trainer)	4.09 ± 1.37		

SE = standard error of estimated coefficient

**Figure 2:**
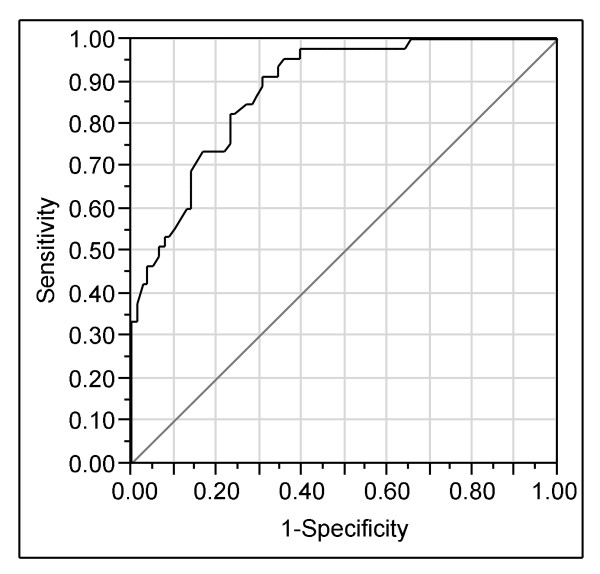
**Receiver operating characteristics (ROC) curve for the logistic regression model predicting no "3-year start"**.

Table 5 presents a logistic regression model predicting horses with no "3-year start". Again, the most important variable was the maternal BLUP index. Horses that had passed a recruitment race also had a markedly increased probability of racing as three year olds. The likelihood of racing in the three year season was decreased by intermediate or hilly pasture topography in the foal season compared to flat. However, a hilly foal pasture was better than a pasture with intermediate topography. As indicated by the interactions, the negative effect of intermediate or hilly foal pasture topography was increased in case of a high dam's index, but decreased with a low dam's index. Further, the positive effect of a hilly pasture compared to intermediate foal pasture topography was slightly reduced in case of a high dam's index and slightly increased in case of a low dam's index. Accustoming the horse to the sulky ("breaking in") before the yearling pasture also slightly reduced the benefit of a high dam's index. The area under the ROC curve for this model is 88.1% (CI 87.7 - 88.5%) (Figure 2).

**Table 5:** Log odds ratio estimates ± standard error, mean values and contributions in the logistic regression model for no "3-year start" and interactions accounted for

	Estimated coefficient ± SE(β_i_)	**Mean value**(${\bar{x}}_{i}$**)**	Contribution (β_i _* ${\bar{x}}_{i}$**)**
**Linear contribution**			
Intercept [0]	81.07 ± 29.99		
Dam's BLUP-index	-0.70 ± 0.27	110.34	-77.24
Hilly or intermediate pasture first summer (1 = yes)	1.57 ± 0.92	0.23	0.36
Hilly pasture first summer (1 = yes)	-1.18 ± 0.64	0.54	-0.64
Recruitment race passed (1 = yes)	-4.39 ± 1.16	0.64	-2.81
			
**Interactions**			
(Dam's BLUP-index - 110.4) * (Hilly or intermediate pasture 1^st ^summer)	0.50 ± 0.28		
(Dam's BLUP-index - 110.4) * (Hilly pasture 1^st ^summer)	0.12 ± 0.13		
(Dam's BLUP-index - 110.4) * (Accustomed to sulky before pasture 2^nd ^summer (1 = yes))	0.17 ± 0.10		

SE = standard error of estimated coefficient

**Figure 3:**
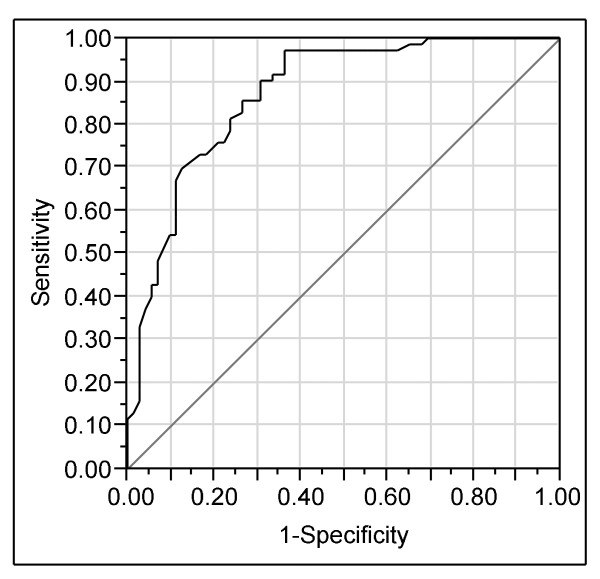
**Receiver operating characteristics (ROC) curve for the logistic regression model predicting no "total start"**.

Table 6 gives a logistic regression model predicting horses with no "total start". As in all models, dam's index was by far the most important variable to predict the dependent variable. Male horses and horses that had passed a recruitment race were more likely to have at least one regular start in the whole study period. The area under the ROC curve for this model is 87.2% (CI 86.7 - 87.7%) (Figure 3).

**Table 6:** Log odds ratio estimates ± standard error, mean values and contributions in the logistic regression model for no "total start"

	Estimated coefficient ± SE (β_i_)	**Mean value**(${\bar{x}}_{\text{i}}$)	Contribution (β_i _*${\bar{x}}_{\text{i}}$**)**
**Linear contribution**			
Intercept [0]	18.35 ± 4.82		
Dam's BLUP-index	-0.14 ± 0.04	110.34	-15.45
Gender (1 = male)	-0.72 ± 0.45	0.46	-0.33
Recruitment race passed (1 = yes)	-3.12 ± 0.57	0.64	-2.00

SE = standard error of estimated coefficient

## Discussion

According to the official NTA race records, 47% of all NSCT horses born in Norway in 2005 passed a recruitment race, and 28% raced in the 3-year season [NTA, personal communication]. Horses that have died are included in these calculations. When adding the 11 horses that had died between birth and start of the study to the included horses, the total group consisted of 155 NSCTs born in 2005. From this group, 92 horses (59%) passed a recruitment race, and 51 horses (33%) raced before end of their 3-year season. The included horses were thereby more successful than average for NSCTs born in 2005. The selection procedure excluded 10% of the reference population (82 horses). The excluded horses were after the least popular sires and the study population therefore represented a biased selection of the reference population. This is the most likely explanation for the described difference in success rate. It is, however, likely that several of the excluded horses were bred mainly for pleasure purposes. Hence, this bias is probably smaller if "NSCT horses bred for racing" is considered to be the true reference population. The selection procedure was therefore considered to be well suited for studies of racehorse performance. With the limited number of sires allowed for breeding, it was not possible to avoid the potential problem that some of the sampled horses were closely related, e.g. half siblings.

A questionnaire-based interview, as used in the present study, has advantages over a written questionnaire. A high compliance level is easy to achieve and misunderstandings can be avoided through two-way communication. As in all interviews and questionnaires, there is a potential for subjective interpretation of both questions and answers. This problem will increase if there is an obvious preferred answer and such questions were largely avoided. Still, different observers may have different opinions of for instance whether a pasture is hilly, intermediate or flat. As all interviews were carried out by the same person, the risk of differences in interpretation of answers was minimised.

The aim of the present study was to identify predictors of early race starts in young NSCT horses. To mainly include early management factors, all variables except those obtained from the NTA were recorded at a single time point around the start of the training period. Later changes to the management were not recorded. As growth rate is individual, any estimation of monthly growth is likely to be incorrect for single horses. The height at 24 months may to some degree reflect early maturity, but will also give an indication of the expected size of the full-grown horse. Individual BLUP estimates were used instead of the actual published BLUP index of included horses, as the updated index includes corrections for individual performances and thereby variables which were to be analysed.

The BLUP index of the dam was included in all three logistic regression models with contributions far superior to all other independent variables in question and was therefore the major predictor of success.

Interestingly, the BLUP estimate and the dam's BLUP index were significantly higher in horses trained before the age of 18 months than for the others. Inherited early maturity and ability to cope with early training might contribute to a high BLUP-index. However, it is also possible that high BLUP-indices are accompanied by high expectations and that higher training efforts are put in horses with high indices.

An interesting question is why the dam's index seems more important than the index of the sire as well as the individual BLUP-estimate. As superior sires are available for all breeders, one might expect a larger variation in dam's indices than in indices of sires. The variations in the indices were however at the same level indicating that also dams are critically evaluated before they are used for breeding. It is possible that the sampling procedure with several horses after the same sire, thereby having identical sire index but different dam indices, may have contributed to this finding. There might, however, also be biological explanations behind this phenomenon. The thought that stamina is inherited through maternal bloodlines has existed for a long time among Thoroughbred breeders [11]. Mitochondrial DNA is inherited solely from the mother and correlation between mitochondrial DNA and racing performances have been demonstrated in Thoroughbreds [11].

It is generally known that intact males are better trotting performers than females [2,12,13]. To compensate this difference, some races are arranged solely for female horses. Still, in our material male horses (intact males and geldings) were more successful than females and it was therefore not surprising that gender entered the logistic regression model for "total start". The reason for this gender difference is probably complex, but differences in temperament may play an important role. Several studies have also demonstrated significant differences in muscle fibre composition between equine sexes [14,15].

High workload during the first months of life is thought to be important for conditioning of the musculoskeletal system [16]. This might explain why pasture topography the first summer enters the logistic regression model for "3-year start", even if the contribution from this variable is small.

A history of disease prior to the moment of examination did influence the probability of passing a recruitment race. It appears reasonable that a diseased young horse may be delayed in development and that this entity has a larger impact on events that take place early in life, such as recruitment races, than on events that take place years later, such as the three and four year seasons. In other words, negative effects can be overcome with time.

Similarly, early professional training did not appear to have any impact on "3-year start" or "total start", but entered the regression model for "recruitment race".

It is hardly surprising that horses not trained by the age of 18 months had a reduced probability for passing a recruitment race. However, according to univariate analysis early training also had a significant impact on "total start", which indicates that early training may be a general benefit for racehorses.

The interactions in the model for "recruitment race" indicate that most of the benefit by professional training was caused by the fact that the horse had actually been trained. If the horse had been trained anyway, the benefit of a professional trainer was considerably reduced.

This also indicates that many amateur trainers posses the necessary skills to train a Coldblooded Trotter and that early start of the training to some extent may compensate a non-professional trainer. NSCTs are not by nature trotters like Standardbreds and must learn the trotting technique through training. Some large individuals tend to struggle more with the trotting technique than smaller ones, which probably explains why the largest horses appeared to benefit more from professional training.

Several superior NSCT horses during the last decades have been comparably small. Some of these horses have been popular breeding objects with high BLUP indices and many NSCT enthusiasts have expressed a concern for a development towards smaller horses. However, univariate analysis results indicate that horses that are large at two years age are more successful for all the dependent variables.

As already discussed, foal pasture topography seems to have an impact on the development and conditioning of a horse. Interactions in the "3-year start" regression model indicate that optimal pasture conditions are more important for horses with low indices. Similarly, early accustoming to the sulky will be more important for a horse with a low index. These findings indicate that less talent to some extent may be compensated by a larger training volume.

The corrections to the model that are caused by these interactions are small compared to the strong contribution from the dam's index. They do, however, indicate that the performance potential of a horse with an undesirable genetic background can be improved by optimal management.

Daily amount of concentrated feeds was significantly different between successful and unsuccessful horses for all three dependent variables in the univariate analyses, but did not enter any of the logistic regression models. This indicates that the daily amount of concentrated feeds was correlated to other variables, such as early training, and that this variable alone is of little importance.

The fact that many horses pass a recruitment race without having any race records in the three or four year seasons may indicate that recruitment race requirements are relatively easy to match. On the other hand, since there are very few active racehorses not having passed a recruitment race, these arrangements seem to have the intended effect, which is to stimulate early training. Passing enters the logistic regression models for both "3-year start" and "total start". Hence, predictors of a passed "recruitment race" indirectly act as predictors for "3-year start" and "total start" even if not included in the respective models.

The nature of biology and genetics imply that superior performers occasionally turn up when least expected. It will therefore never be possible to construct a perfect model for prediction of the racing career in a single horse. The results in this study confirm the importance of a superior pedigree, but the path leading to a good performing racehorse will always be long and difficult.

Too strong focus on genetic traits, especially in limited populations, bears with it a risk of increasing inbreeding tendencies. The inbreeding coefficients have been rising within the NSCT breed over the last decades [17]. This tendency gives rise to some concern, but is monitored closely.

Some horse owners fear that early training may compromise the longevity of the horse. This question can not yet be evaluated in these study objects. By re-evaluating the racing and performance records of the included horses in a few years, the long term effects of the recorded independent variables can be investigated. Possible explanations for the apparent difference between impact of parental indices should be investigated further.

## Conclusions

The results of the present study confirm the value of the BLUP index as an important tool for the NSCT breeding program. Very few horses start in regular trotting races as three or four year olds without a record of a passed recruitment race. Therefore these arrangements appear to stimulate early training as intended. All other included independent variables are comparably weak predictors of starting in recruitment races as well as early regular races.

## Competing interests

The authors declare that they have no competing interests.

## Authors' contributions

All authors participated in design of the study. TR performed all the interviews and examinations of the included horses, and drafted the manuscript. CFI helped to draft the manuscript. SL was responsible for the statistical analysis. All authors read and approved the final manuscript.
